# A portable system to measure knee extensor spasticity after spinal cord injury

**DOI:** 10.1186/s12984-024-01326-9

**Published:** 2024-04-09

**Authors:** Dalia De Santis, Monica A. Perez

**Affiliations:** 1 Shirley Ryan Abilitylab, Chicago, IL 60611 USA; 2 Department of Physical Medicine and Rehabilitation, Northwestern University, Chicago, IL 60611 USA; 3 Edward Hines Jr. VA Hospital, Hines, IL 60141 USA

**Keywords:** Spasticity, Spinal cord injury, Pendulum test, Modified Ashworth Scale, Inertial measurement units, Reliability

## Abstract

**Background:**

The pendulum test is a quantitative method used to assess knee extensor spasticity in humans with spinal cord injury (SCI). Yet, the clinical implementation of this method remains limited. The goal of our study was to develop an objective and portable system to assess knee extensor spasticity during the pendulum test using inertial measurement units (IMU).

**Methods:**

Spasticity was quantified by measuring the first swing angle (FSA) using a 3-dimensional optical tracking system (with external markers over the iliotibial band, lateral knee epicondyle, and lateral malleolus) and two wireless IMUs (positioned over the iliotibial band and mid-part of the lower leg) as well as a clinical exam (Modified Ashworth Scale, MAS).

**Results:**

Measurements were taken on separate days to assess test–retest reliability and device agreement in humans with and without SCI. We found no differences between FSA values obtained with the optical tracking system and the IMU-based system in control subjects and individuals with SCI. FSA values from the IMU-based system showed excellent agreement with the optical tracking system in individuals with SCI (ICC > 0.98) and good agreement in controls (ICC > 0.82), excellent test–retest reliability across days in SCI (ICC = 0.93) and good in controls (ICC = 0.87). Notably, FSA values measured by both systems showed a strong association with MAS scores ($\uprho$ ~ −0.8) being decreased in individuals with SCI with higher MAS scores, reflecting the presence of spasticity.

**Conclusions:**

These findings suggest that our new portable IMU-based system provides a robust and flexible alternative to a camera-based optical tracking system to quantify knee extensor spasticity following SCI.

## Introduction

Spasticity is a common symptom present in a large number of individuals with spinal cord injury (SCI) [1, 2]. Despite having a considerable impact on independence and quality of life after SCI [3, 4], its quantification remains limited. Clinical exams, such as the Modified Ashworth Scale (MAS) [5] and the Tardieu scale [6], use nominal scales for the quantifications of spasticity and have limited validity and reliability [7–11]. Mechanical devices used to quantify resistance to a passive stretch at controlled amplitudes and velocities need expensive and bulky equipment and therefore are less suitable for routine use in clinical environments [12]. Thus, there is a pressing need for developing objective and portable systems to measure spasticity [13–15, 2, 16].

The goal of our study was to develop a system to assess knee extensor spasticity, which is commonly observed in individuals with SCI [1, 4]. At present, the pendulum test is a widely used biomechanical test for evaluating knee extensor spasticity using kinematic analysis [17]. The test quantifies the effect of a gravity-induced stretch of the knee extensor muscle on the leg kinematics and is performed with the participant sitting or supine with the legs hanging over the edge of a table. The operator first brings the leg to full extension and then suddenly releases it letting the limb swing under the action of gravity. A reduction of the first swing motion of the leg (first swing angle, FSA) has been associated with increased stretch reflex activity [18–21]. The pendulum test has been validated in controls [22] and has a high test–retest reliability and sensitivity to detect variations in spasticity in humans with SCI [20, 23–25], correlating with clinical scores [26–28]. Although, the pendulum test has been widely used alongside clinical scales barriers remain to implementing this exam in the clinic [13, 16, 29, 30]. Over the years, the pendulum test has been instrumented using video recordings [22], electrogoniometers [31, 32], gyroscopes [28], and accelerometers [33] making comparisons and standardization across outcomes difficult. Video recordings and systems based on optical markers are considered the gold standard for administering the pendulum test in laboratory environments but the need for markers to be visible at all times complicates physical examinations and measurements are sensitive to soft tissue artifacts and errors in markers positioning [34]. Electrogoniometers require positioning on two segments of a joint and are sensitive to improper alignment with the joint axes [35]. Gyroscopes can lead to overestimation of joint angles at higher angular speeds and accuracy degrades with time, while estimations from accelerometers are affected by measurement noise and integration drift [36]. Inertial measurements units (IMUs) have been extensively adopted as a reliable and inexpensive alternative to video recordings and systems based on optical markers for estimating lower limb kinematics [37–41]. We hypothesized that a portable system using two wireless IMUs would have good reliability in assessing knee extensor spasticity during the pendulum test as a 3-dimensional optical tracking system.

To address this question, we evaluated FSA values obtained with an optical tracking system and an IMU-based system in individuals with and without SCI on two different days. FSA values were compared with the MAS in individuals with SCI.

## Materials and methods

### Subjects

Twenty-three individuals with SCI (mean age 49.7 ± 13.4 years, 8 female; Table 1) and 22 age-matched controls (mean age 43.9 ± 16 years, 12 females, p = 0.2) participated in the study. All participants gave informed consent to the experimental procedures, which were approved by the Northwestern University Institutional Review Board and performed in accordance with the Declaration of Helsinki. Individuals with SCI were included in the study if they had sustained a chronic injury (≥ 1 year) at or above T12 and had no concurrent orthopedic conditions limiting the range of motion at the knee or affecting the passive movement of the leg. Twelve individuals were under antispasmodic medications or GABA-derivative drugs at the time of enrollment (Table 1). These individuals were asked to withhold the medication on the day of the test to control the potential influence of these medications on the results of the assessment.

**Table 1:** Spinal cord injury participants

Participant	Sex	Age (years)	Years after injury	Etiology	Level	AIS score	Spasticity medication	MAS rating			
								Day 1		Day 2	
								R	L	R	L
001	M	54	1	T	T12	D	*None*	0	0	0	0
002	M	52	3	T	C7	C	*None*	1 +	1 +	1 +	1 +
003	F	66	6	T	T4	D	BAC	3	3	3	3
004	M	68	33	T	C8	C	*None*	0	0	0	0
005*	M	59	36	T	C5	B	*None*	–	1 +	–	1 +
006	F	47	27	NT	T4	C	BAC	3	4	3	4
007*	M	57	38	T	T10	A	BAC, TIZ	–	0	–	0
008	F	39	15	NT	L3	A	BAC	0	0	0	1
009	F	49	16	T	C5	B	*None*	1	1 +	0	0
010	M	33	16	T	T4	C	*None*	0	0	0	0
011	F	52	18	NT	T10	D	BAC, TIZ, GBP	4	3	4	2
012	M	58	3	NT	C4	D	*None*	1	1 +	1 +	1 +
013	M	61	8	NT	C4	D	*None*	1 +	1 +	0	0
014	M	35	15	T	T10	A	None	2	1 +	2	1 +
015	M	48	27	T	T5	A	BAC	3	3	3	3
016	M	68	4	T	T5	A	BAC	0	0	0	1
017	M	33	8	T	C8	C	BAC	0	1 +	0	1 +
018	F	33	11	T	C1	D	*None*	2	3	2	3
019	F	45	10	NT	C2	D	BAC	3	3	4	3
020	F	48	2	T	T3	D	BAC	3	2	2	2
021**	M	21	5	T	C6	C	BAC	2	3	–	–
022**	M	42	18	T	C2	D	*None*	2	0	–	–
023**	M	74	3	T	C4	D	BAC, TIZ, GBP	1 +	1 +	–	–

*M* male, *F* female, *AIS* American Spinal Injury Association Impairment Scale, *T* traumatic, *NT* non-traumatic, *MAS* Modified Ashworth Scale, *BAC* baclofen, *GBP* gabapentin, *TIZ* tizanidine

^*^R leg not tested due to physical constraints

^**^Participated in only one study session

### Experimental procedures

**Fig. 1:**
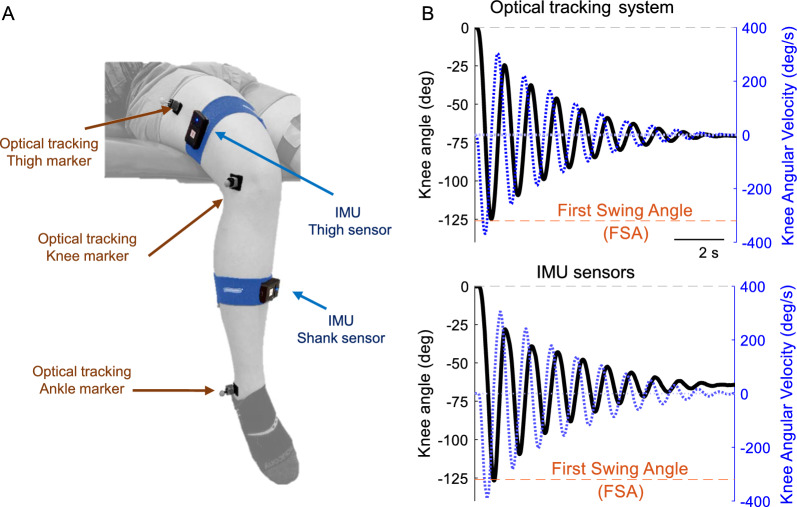
Experimental setup. **A** Positioning of the leg IMU-based sensors on the thigh and shank using adhesive velcro straps and of the passive markers for the optical tracking system on the anatomical landmarks. **B** Example of knee kinematics during the pendulum test. The knee angle is depicted in black, while the knee angular velocity is in blue. The arrows indicate the measurement corresponding to the first swing angle (FSA)

Spasticity in the quadriceps femoris muscle was examined in SCI participants through the MAS and the pendulum Test. The two tests were repeated on two separate days at the same time of the day. All control individuals and 20 SCI individuals participated in two study sessions a week apart, while 3 SCI individuals participated only in one session. Assessments were carried out by a single assessor on both legs. The MAS was performed before the pendulum test separated by a minimum of 5 min. Because several studies have shown that knee extensor spasticity is increased when tested in a supine or semi-supine position compared to upright sitting [18, 19, 21, 42], both assessments were carried out with the participant lying in a semi-supine position on a table with the torso supported at a 30° of flexion by a therapy wedge and the head resting on a pillow. The legs were hanging off the table maintaining a distance of 2 inches between the popliteal fossa and the edge of the table (Fig. 1A). This position was preferred over a supine position to maximize patient’s comfort while ensuring reliable measurements of spasticity.

*MAS*. This clinical scale measures resistance encountered during manual passive muscle stretching using a six-point ordinal scale (0 = no increase in tone, 1/ + 1 = slight increase in tone with a catch and release or minimal resistance at the end or less than half of the range of movement, respectively, 2 = more marked increased tone through most of the range of movement but affected part easily moved, 3 = considerable increase in tone and passive movement difficult, and 4 = affected part rigid) [5]. Before performing the assessment, the knee joint was first slowly moved from full extension to full flexion through the available range. Subsequently, the leg was flexed from full extension to the maximum flexion within 1 s. The MAS scores from knee extensors were acquired bilaterally.

### Pendulum test

The test started with the participant fully relaxed with both legs hanging over the edge of a padded therapy table (Fig. 1A). Then, the examiner held the subject’s heel and slowly extended the leg to the horizontal position while the subject was instructed to relax. After a few seconds, the heel was released, and the leg was allowed to swing freely under the action of gravity until it came to a complete rest. The pendulum test was repeated 10 times per leg with a minimum of 30 s rest between repetitions. Knee kinematics was recorded simultaneously with an optical tracking system using passive markers and a portable IMU-based system.

### Optical tracking system

The optical motion capture system consisted of 6 infrared cameras (Flex3, OptiTrack, Natural Point, Inc.) positioned at a distance of ~ 2.5 m from the participant and calibrated to yield a tracking precision < 1 mm. Three 6-mm reflective markers were attached on the leg over the halfway point of the iliotibial band (Thigh marker, Fig. 1A), the lateral knee epicondyle (Knee marker, Fig. 1A), and the lateral malleolus (Ankle marker, Fig. 1A). The 3-marker configuration was chosen as it has been most commonly adopted in the literature when evaluating spasticity during the pendulum test [1, 24, 27, 43, 44]. The 3-dimensional coordinates of the markers were sampled at 100 Hz and pre-processed using the Motive 2.0 software (Natural Point, Inc.), and the data was exported for offline analysis.

### IMU-based system

The system consisted of 2 wireless IMUs (3-Space™ Wireless, YostLab Inc.) with tri-axial accelerometers (± 12 g), compass (± 1.30 Gauss) and gyroscope (± 2000º/s) sensors. The accelerometer and compass sensors were calibrated following a gradient descent procedure prior to the test and their orientation relative to a common reference frame was recorded. Before starting the data collection, the gyroscope was calibrated after positioning the IMUs on a level surface. Subsequently, the sensors were attached to the thigh (IMU Thigh sensor, Fig. 1A) and the shank (IMU Shank sensor, Fig. 1A) of the participant by means of Velcro® pads onto two straps (Fabrifoam NuStim Wrap) positioned 5 inches above the top of the patellar bone and 5 inches below the head of the fibula. The thigh sensor was positioned with the y axis parallel to the axis of the thigh and the shank sensor was attached on the shank with the y axis parallel to the axis of the tibia. The location of the sensors was chosen to minimize the effect of soft tissue movement on the orientation estimates during the test [45]. The quaternion orientation of each sensor in space expressed in the common frame of reference was computed via a proprietary quaternion-based sensor fusion gradient descent algorithm (QGRAD2™ fusion firmware) running on board of each sensor at an update rate of 312 Hz (3.2 ms update interval). Since the tests were conducted within a clinical environment with uncontrollable sources of magnetic disturbance, the compass sensor was switched off during the recordings. The orientation, acceleration, and angular velocity data from the two sensors was wirelessly transmitted to a computer running a custom C +  + application and saved for offline analysis.

### FSA

The knee angle trajectory during the pendulum test recorded by the motion capture system and the IMU-based system was computed through a custom Matlab® code (Matlab R2020a, Mathworks™). The IMU-based was first aligned in time and resampled at a frequency of 100 Hz using a shape-preserving piecewise cubic function. The orientation of the shank sensor relative to the thigh sensor was computed at every sample $i$ as follows:

1 $${q}_{rel}\left(i\right)={q}_{shank}\left(i\right)\otimes {q}_{thigh}^{-1}\left(i\right),$$

where $\otimes$ stands for quaternion multiplication and ${q}_{thigh}^{-1}$ is the inverse/conjugate quaternion. The relative orientation was then referred to the orientation at the instant of full extension preceding the leg drop, ${i}^{*}$:

2 $${\widehat{q}}_{rel}\left(i\right)={q}_{rel}\left(i\right)\otimes {q}_{rel}^{-1}\left(i={i}^{*}\right).$$

The instant ${i}^{*}$ was defined through visual inspection of the angular velocity of the shank sensor and corresponded to an instant when the leg was stationary in extension (i.e. the norm of gyroscope data from the shank sensor was approximately zero). The knee angle was then computed from the relative quaternion as the arctangent between its imaginary $\left( {\hat{q}_{{rel_{x} }} ,\,\hat{q}_{{rel_{y} }} ,\,\hat{q}_{{rel_{z} }} } \right)$ and real $\left( {\hat{q}_{{rel_{w} }} } \right)$ components:

3 $$\theta_{IMU} = 2*{\text{atan}}2\left( {\left\| {\hat{q}_{{rel_{x} }} ,\,\hat{q}_{{rel_{y} }} ,\,\hat{q}_{{rel_{z} }} } \right\|,\,\hat{q}_{{rel_{w} }} } \right)$$

Following these computations, the knee angle at full extension corresponded to a value of 0°, while negative (positive) angles corresponded to flexion of the right (left) knee. The knee flexion angle from the optical tracking system in a 3-marker setting is conventionally computed as the angle between the vectors aligned with the proximal ($\overrightarrow{\text{Knee-Thigh}}$) and distal segments $(\overrightarrow{\text{Knee-Ankle}}$). In our case, however, this computation led to underestimation of the knee angle of approximately 20% on average as compared to the IMU-based system. Thus, we chose to approximate the knee flexion angle with the angle spanned by the segment joining the 3-dimensional coordinates of Knee and Ankle sensors as the leg moved from full extension (angle of 0°) through flexion after being released by the assessor:

4 $${{\varvec{M}}}_{23}(i)={\varvec{A}}{\varvec{n}}{\varvec{k}}{\varvec{l}}{\varvec{e}}\left(i\right)-{\varvec{K}}{\varvec{n}}{\varvec{e}}{\varvec{e}}\left(i\right)$$

5 $${\theta }_{OTS}={\text{atan}}2\left(\Vert {{\varvec{M}}}_{23}\left(i\right)\times {{\varvec{M}}}_{23}(i={i}^{*})\Vert ,{{\varvec{M}}}_{23}\left(i\right)\cdot {{\varvec{M}}}_{23}(i={i}^{*})\right)$$

Knee angle trajectory and angular velocity were recorded during the pendulum test using the motion capture system and the IMU-based system and the respective angular velocity (Fig. 1B). The FSA was defined as the angle in degrees at which the swinging leg first reversed direction from flexion to extension (i.e. the angle at which the velocity of the leg first becomes zero). FSA values were computed on individual knee angle trajectories obtained from Eqs. (3) and (5) and averaged over the 10 repetitions to obtain a single value per participant with each of the techniques. The within-subject variability in FSA values was also computed as the standard deviation of the repeated measurements within each session. As there is no evidence for dominance effects on limb responses to the pendulum test [22], responses from dominant and non-dominant limbs were treated as independent. The angle at peak speed was defined as the knee angle at the time instant of peak angular speed, when the absolute velocity during the first leg swing was maximal. In addition, we examined the variability between trials computing the IMU drift in Eq. (6) as the error between the IMU and optical tracking system in each trial relative to the first trial in absolute value:

6 $$\begin{array}{*{20}c} {error\left( n \right) = FSA_{{IMU}} \left( n \right) - FSA_{{OTS}} \left( n \right),\,n = 1, \ldots 10} \\ {drift\left( m \right) = abs\left( {error(m) - error(1)} \right),\,m = 2, \ldots 10} \\ \end{array}$$

*Data analysis.* Normality of FSA values was tested with the Shapiro–Wilk’s test and homogeneity of variances with the Levene’s test of equality, and Mauchly’s test of sphericity. When the sphericity assumption was not met, the Greenhouse–Geisser correction was applied. The Mann–Whitney U test and the median test for independent samples were used to assess the effect of GROUP (controls, SCI) on the average FSA values obtained with either SYSTEM (optical tracking system, IMU-based system). We further evaluated if multiple repetitions of the test impacted the variability across measurements obtained with the two systems. Repeated measures analysis of covariance (ANCOVA) with the FSA value obtained on trial 1 as covariate was used to assess the effect of REPETITION (2 to 10) and DAY (Day 1, Day 2) as within factors and GROUP as between factor on the FSA values obtained with the two systems. A repeated measures ANOVA was performed to assess the effect of SYSTEM as between factor and DAY as within factor on the average FSA values. As the data from control and SCI participants differed in terms of variance, the analysis was performed separately for each group.

A repeated measures ANOVA was performed to assess the effect of GROUP as between factor and DAY as within factor on the average absolute difference between measures obtained with both systems (referred as ‘absolute error’). In addition, a repeated measures ANOVA with Bonferroni corrected post-hoc comparisons was used to assess the effect of REPETITION on drift measurements. As we assumed drift to be independent of the group and day of test, measurements across group and days have been combined.

A one-way ANOVA was performed to assess the effect of a grouping based on MAS ratings (spastic: MAS $\ge$ 2, mild/no spasticity: MAS < 2, controls) on the average FSA values and the angle at peak speed obtained on Day 1. The same analysis was repeated for Day 2. Bonferroni post hoc analysis was used to test for significant comparisons across groups. Intra-class correlation coefficients (ICC) (2,1) and their 95% confidence intervals were calculated based on a single-measurement, 2-way mixed-effects model [46]. ICC was defined as excellent ≥ 0.90, good ≥ 0.75, and moderate to poor < 0.75 [47]. Absolute agreement and consistency were evaluated on the average FSA values in 22 controls and 23 individuals with SCI obtained from the two systems in a single study session. Test–retest reliability for each system was quantified by measuring the absolute agreement of FSA values on two separate study sessions. Test–retest reliability for MAS was assessed by the Cohen’s $\kappa$ coefficient. Spearman correlation was used to compare MAS and FSA values across days and systems. All statistical analyses were conducted using SPSS statistical package version 26 (SPSS Inc, Chicago, IL) and the significance was set to 0.05.

## Results

### FSA

**Fig. 2:**
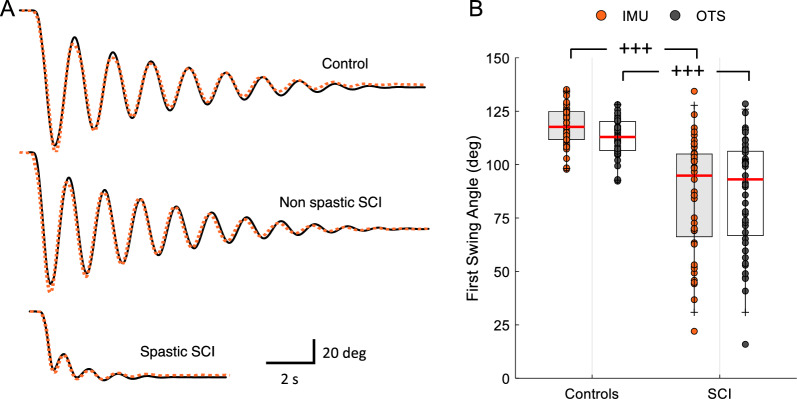
First swing angle (FSA) between groups. **A** Representative traces of the knee angle obtained with the IMU-based system (orange) and the optical tracking system (black) during the pendulum test in a control individual (top traces), an individual with SCI without spasticity (middle traces) and an individual with SCI with spasticity (bottom traces). **B** The box plot charts show the FSA values obtained using the optical tracking system (OTS, gray) and the IMU-based system (IMU, orange) in both groups. The abscissa shows the group tested (controls, SCI) and the ordinate shows the FSA values in degrees. The top and bottom lines of the box indicate the 75th percentile (top quartile) and 25th percentile (bottom quartile), respectively. The red lines in the middle of the boxes indicate the 50th percentile (median). The two bars extend from the maximum and minimum values. Range. ***p < 0.01

Figure 2A shows kinematic traces recorded with the optical tracking (black solid lines) and the IMU-based (orange dotted lines) system during the pendulum test in one control subject and 2 participants with SCI. Note that the FSA was decreased in the participant with SCI with spasticity (lower traces) compared to the participant without spasticity (middle traces) and the control subject (upper traces). The Mann–Whitney U test showed an effect of GROUP on FSA values obtained on Day 1 with the optical tracking (U = 302, n_1_ = 43, n_2_ = 44, p < 0.001) and the IMU-based (U = 186, n_1_ = 43, n_2_ = 44, p < 0.001; Fig. 2B) systems.

ANCOVA test showed no effect of REPETITION (${F}_{\mathrm{2,167}}$ = 0.2, p = 0.8, ${\eta }_{p}^{2}$ = 0.003), DAY (${F}_{\mathrm{1,69}}$ = 0.03, p = 0.8, ${\eta }_{p}^{2}$ < 0.001), GROUP (${F}_{\mathrm{1,69}}$ = 2.5, p = 0.1, ${\eta }_{p}^{2}$ = 0.03) or their interaction (REPETITION × DAY: ${F}_{\mathrm{4,280}}$ = 0.5, p = 0.7, ${\eta }_{p}^{2}$ = 0.008; REPETITION × GROUP: ${F}_{\mathrm{2,167}}$ = 0.8, p = 0.4, ${\eta }_{p}^{2}$ = 0.01) on FSA values obtained using the optical tracking system. Similarly, no effect of REPETITION (${F}_{\mathrm{2,131}}$ = 0.4, p = 0.6, ${\eta }_{p}^{2}$ = 0.007), DAY (${F}_{\mathrm{1,63}}$ = 0.02, p = 0.9, ${\eta }_{p}^{2}$ < 0.001), GROUP (${F}_{\mathrm{1,63}}$ = 1.0, p = 0.3, ${\eta }_{p}^{2}$ = 0.01) or their interaction (REPETITION × DAY: ${F}_{\mathrm{5,310}}$ = 0.6, p = 0.7, ${\eta }_{p}^{2}$ = 0.009; REPETITION × GROUP: ${F}_{\mathrm{2,131}}$ = 0.3, p = 0.6, ${\eta }_{p}^{2}$ = 0.006) was found on FSA values with the IMU system. Altogether, this analysis suggest that it is less likely that multiple repetitions of the test contributed to FSA measures.

**Fig. 3:**
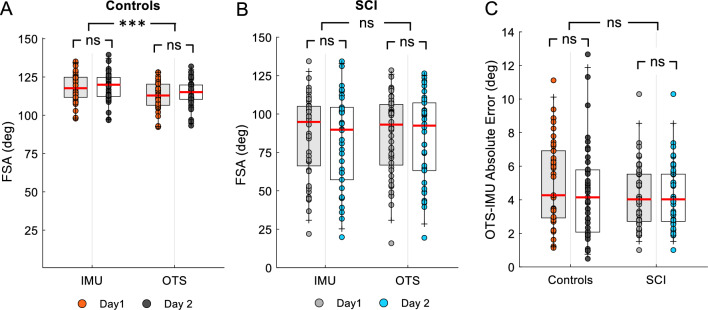
First swing angle (FSA) between days and systems. The box plot charts show the FSA values obtained using the optical tracking system (OTS) and the IMU-based system (IMU) in controls (**A**) and SCI (**B**) participants. The abscissa shows the system tested (OTS, IMU) on Day 1 and Day 2 (A, controls: Day 1 = orange, Day 2 = gray; B, SCI: Day 1 = light gray, Day 2 = light blue) and the ordinate shows the FSA values in degrees. **C.** The box plot chart shows the difference in absolute FSA values (refereed as ‘absolute error’) obtained using both systems in both groups. The abscissa shows the group tested (controls, SCI) and the ordinate shows the absolute error in degrees. In all graphs, the top and bottom lines of the box indicate the 75th percentile (top quartile) and 25th percentile (bottom quartile), respectively. The red lines in the middle of the boxes indicate the 50th percentile (median). The two bars extend from the maximum and minimum values. Range. ***p < 0.001

Repeated measures ANOVA showed an effect of SYSTEM (${F}_{\mathrm{1,84}}$= 5.4, p < 0.02, ${\eta }_{p}^{2}=0.9$), but not DAY (${F}_{\mathrm{1,84}}$= 3.0, p = 0.08, ${\eta }_{p}^{2}=0.04$), or of their interaction (${F}_{\mathrm{1,84}}$= 1.3, p = 0.2, ${\eta }_{p}^{2}=0.1$) on FSA values in control participants (Fig. 3A). The IMUs measured FSA values that were 4.3° ± 3.1° larger than the optical tracking system. The within-subject variability in control subjects was 3.1° (min = 1.0°, max = 7.2°) and 3.4° (min = 1.1°, max = 7.6°) for the optical tracking system and for IMU-based system, respectively. In SCI participants, repeated measures ANOVA showed no effect SYSTEM (${F}_{\mathrm{1,74}}$= 0.34, p = 0.8, ${\eta }_{p}^{2}<0.001$), DAY (${F}_{\mathrm{1,74}}$= 0.7, p = 0.3, ${\eta }_{p}^{2}=0.01)$ or their interaction (${F}_{\mathrm{1,74}}$= 0.07, p = 0.7, ${\eta }_{p}^{2}=0.001$) on FSA values (Fig. 3B). FSA values obtained in the SCI group were more broadly distributed reflecting the presence of individuals with and without spasticity, ranging between 22.0 and 134.3° on Day 1 with the IMU-based system and 15.9–128.4° on Day 1 using the optical tracking system. Similarly, in Day 2, FSA values obtained using the IMU-based system ranged between 19.8 and 134.3°and between 19.4 and 126.4° using the optical tracking system. Note that the within-subject variability in SCI participants was 5.0° (min = 1.5°, max = 20.6°) and 5.3° (min = 1.5°, max = 19.7°) for the optical tracking system and for IMU-based system, respectively.

Figure 3C illustrates the absolute error between FSA values measured by the optical tracking system and the IMU-based system on different days in control and SCI participants. Repeated measures ANOVA showed no effect of DAY (${F}_{\mathrm{1,79}}$= 0.009, p = 0.9, ${\eta }_{p}^{2}<0.001$), or GROUP (${F}_{\mathrm{1,79}}$= 0.89, p = 0.34, ${\eta }_{p}^{2}=0.01$) but of their interaction (${F}_{\mathrm{1,79}}$= 4.0, p = 0.04, ${\eta }_{p}^{2}=0.4$) on the absolute error. The difference in FSA values between systems was 4.7° ± 2.6° in controls (Day 1 = 5.1° ± 2.5° range 1.1° to 11.1°, Day 2 = 4.4° ± 2.7° range 0.5° to 12.7°) and 4.5° ± 2.4° in SCI (Day 1 = 4.2° ± 1.8° range 1.0° to 10.3°, Day 2 = 4.8° ± 2.8° range 1.0° to 11.4°).

Repeated measures ANOVA showed a significant effect of REPETITION on drift measures (${F}_{\mathrm{5,903}}$ =9.9, p < 0.001, ${\eta }_{p}^{2}$ = 0.06). *Post-hoc* analysis revealed that the effect of drift on the measurements was significant from the 7th repetition onward. The average change in drift estimates was 0.8° at trial 7 (p = 0.015, 95% CI 0.08–1.53°), 1.2° at trial 8 (p = 0.001, 95% CI 0.3–2.1°), 1.6° at trial 9 (p < 0.001, 95% CI 0.6–2.6°) and 1.2° at trial 10 (p = 0.002, 95% CI 0.3–2.0°).

**Fig. 4:**
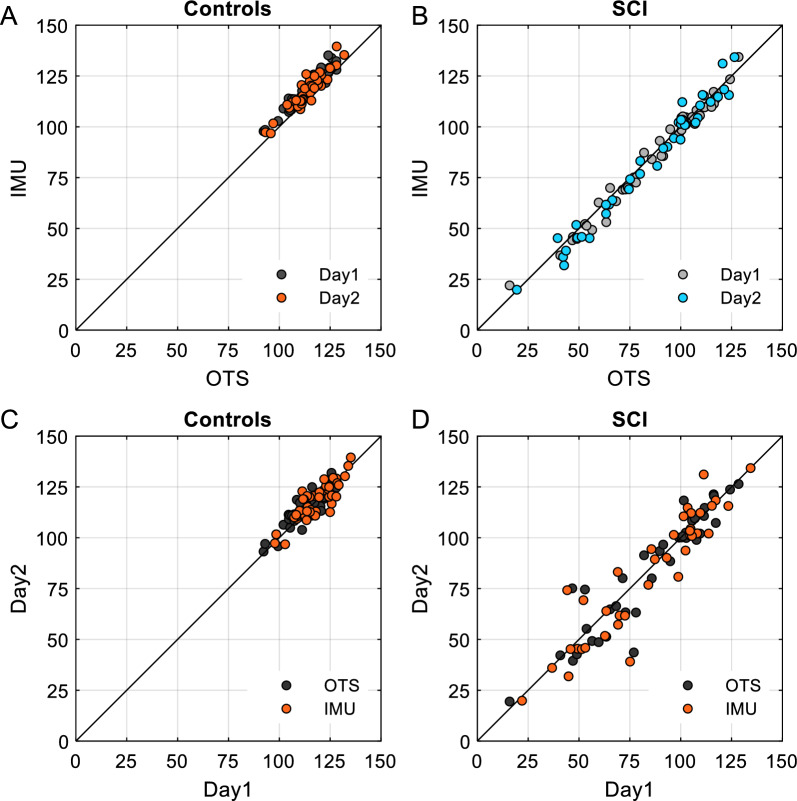
Correlations. **A**, **B** Device agreement between IMU and optical tracking system in control (**A**) and SCI (**B**) individuals. **C**, **D** Test–retest reliability of the average FSA measurements obtained on Day 1 and Day 2 in control (**C**) and SCI (**D**) individuals. The solid line represents equality between the two measurements. Each dot represents the average FSA measurement obtained on one limb for one individual

Table 2 shows the results of device agreement in controls and SCI participants measured on Day 1 and Day 2. Note that on both days there was an excellent to good agreement between FSA values obtained by the optical tracking system and the IMU-based system in controls (Fig. 4A) and individuals with SCI (Fig. 4B). In addition, Table 3 shows the results of the test–retest reliability between Day 1 and Day 2 FSA values for the optical tracking system and the IMU-based system in both groups. We found an excellent to good agreement between FSA values obtained on different days by both systems in controls (Fig. 4C) and SCI (Fig. 4D) participants.

**Table 2:** Reliability analysis: device agreement

	Absolute agreement		Consistency		ANOVA		
	ICC (2,1)	95% CI	ICC (2,1)	95% CI	F	dof	p-value
Control							
Day 1	0.829	− 0.033 to 0.954	0.952	0.913–0.974	40.6	42	< 0.001
Day 2	0.854	0.229 to 0.952	0.930	0.874–0.961	27.5	42	< 0.001
SCI							
Day 1	0.988	0.979 to 0.994	0.989	0.980–0.994	179.3	43	< 0.001
Day 2	0.992	0.984 to 0.996	0.992	0.984–0.996	130.4	37	< 0.001

*ICC* intraclass correlation coefficient, *OTS* optical tracking system, *CI* confidence interval, *dof* degrees of freedom

**Table 3:** Reliability analysis: test–retest absolute agreement

	ICC (2,1)	95% CI	F	dof	p-value
Control					
OTS	0.876	0.775–0.933	16.5	42	< 0.001
IMU	0.864	0.763–0.924	13.5	42	< 0.001
SCI					
OTS	0.932	0.874–0.964	27.9	37	< 0.001
IMU	0.925	0.861–0.960	25.3	37	< 0.001
Overall					
OTS	0.952	0.927–0.969	40.4	80	< 0.001
IMU	0.951	0.925–0.968	39.8	80	< 0.001

*ICC* intraclass correlation coefficient, *OTS* optical tracking system, *IMU*  inertia motion unit based system, *CI* confidence interval, *dof* degrees of freedom

### MAS

**Fig. 5:**
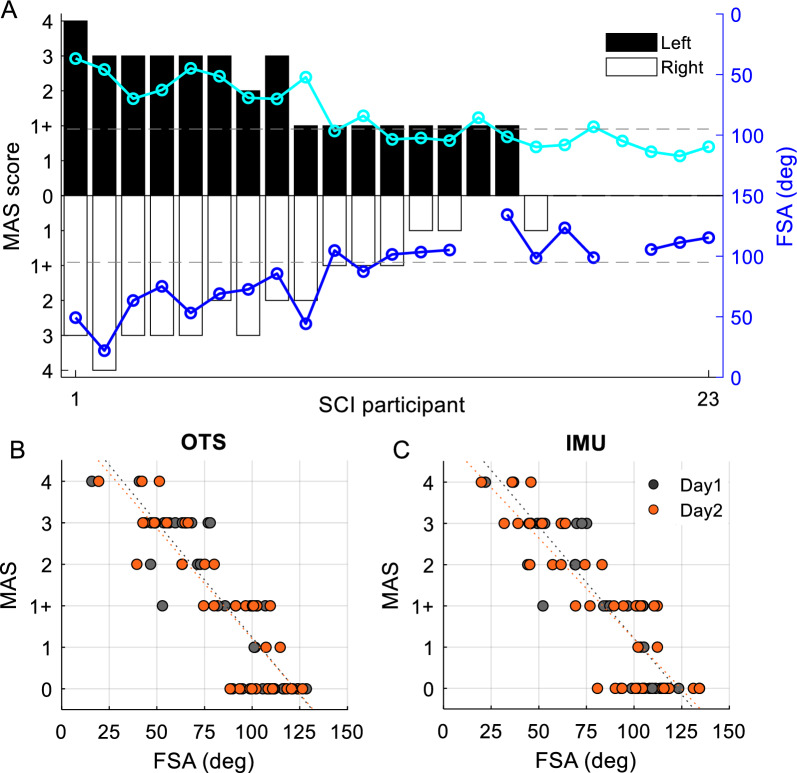
Modified Ashworth Scale (MAS) and First swing angle (FSA) values. **A** MAS scores obtained on Day 1 for the right (white bars) and left (black bars) leg for individuals in the SCI group. The FSA values for each participant are indicated by the blue and cyan circles on each side, respectively. The dotted gray lines represents the minimum FSA value obtained in control individuals. The graphs show the relationship between MAS scores and FSA values on Day 1 (orange dots) and Day 2 (black dots) using the optical tracking system (OTS) (**B**) and the IMU-based system (IMU) (**C**). The abscissa shows the FSA values in degrees and the ordinate shows the MAS scores. Each dot represents the average FSA value and MAS scores from a single limb. The dotted line indicate the least square linear regression between the MAS and FSA values on each day

The MAS scores for each SCI participant are reported in Table 1. Figure 5A shows the bilateral distribution of the MAS scores and FSA values obtained by the IMU-based system on Day 1 in individuals with SCI. The horizontal dotted lines represent the lower bound of FSA values in controls. Cohen’s $\kappa$ showed that MAS scores between days of test have moderate test–retest reliability (Cohen’s $\kappa$ = 0.7). The MAS scores and FSA values show a very strong Spearman’s correlation when measures were obtained with optical tracking system (Day 1: $\rho \left(44\right)=-0.87, p<0.001$; Day 2: $\rho \left(38\right)=-0.85, p<0.001$; Fig. 5B) and IMU-based system (Day 1: $\left(44\right)=-0.89, p<0.001$; Day 2:$\rho \left(38\right)=-0.84, p<0.001$; Fig. 5C). Note that FSA values were between 12 and 34° in individuals with MAS = 4 and between 18–73° in individuals with MAS = 3. These values largely overlap with FSAs measured in individuals with MAS = 2 (24–85°) and MAS = 1 + (42–105°). We also found that the knee angle followed a dampened pendular motion even in the more severely spastic individuals, consistent with the effect of gravity on the leg mass. Overall, this analysis suggest that it is less likely that pendulum test measurements obtained in people with MAS 3 or 4 were impacted by severe rigidity.

Another measurement that can contribute to the assessment spasticity during the pendulum test is angle at peak speed of the knee during flexion. Similar to FSA values, we found that the angle at peak speed computed with the IMU-based system was strongly correlated MAS scores (Day 1: $\rho \left(44\right)=-0.84, p<0.001$; Day 2: $\rho \left(38\right)=-0.76, p<0.001$). The angle at peak speed was 60.1º in controls (min = 43.3º/s, max = 76.5º) on Day 1 and 60.0º (min = 46.0º, max = 73.4º) on Day 2, while in SCI participants was 45.4º (min = 10.5º, max = 82.1º) on Day 1 and 43.4º (min = 7.9º, max = 66.9º) on Day 2. Repeated measured ANOVA showed on effect of GROUP (${F}_{\mathrm{1,75}}$=$33.35$, p < 0.001, ${\eta }_{p}^{2}=0.31$), but not DAY (${F}_{\mathrm{1,75}}$ = 1.72, p = 0.19, ${\eta }_{p}^{2}=$ 0.02), or their interaction (${F}_{\mathrm{1,75}}$= 1.27, p = 0.26, ${\eta }_{p}^{2}=0.02$) on the angle at peak speed recorded by the IMU system. The angle at peak speed was on average 15.6º (26%) larger in controls than individuals with SCI, indicating a possible influence of individuals with spasticity on the measurement. In addition, the peak speed was 355.6º/s in controls (min = 293.3º/s, max = 406.6º/s) on Day 1 and 363.4º/s (min = 293.8º/s, max = 411.6º/s) on Day 2. In SCI participants the peak speed was 324.1º/s (min = 135.7º/s, max = 426.9º/s) on Day 1 and 332.1º/s (min = 161.7º/s, max = 444.2º/s) on Day 2.

**Fig. 6:**
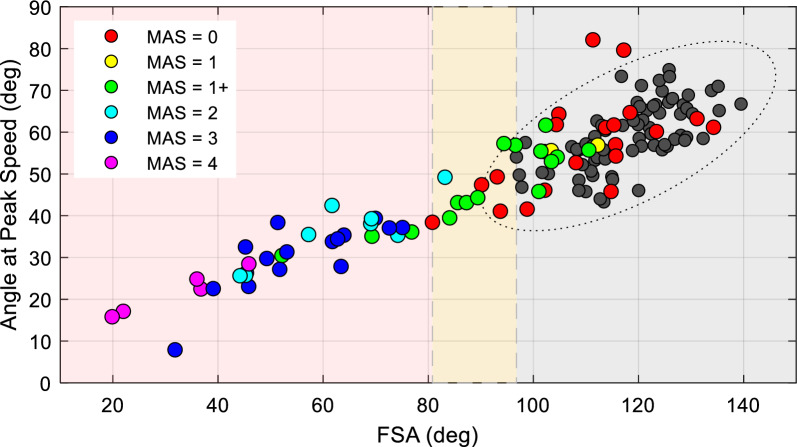
Relationship between FSA, angle at peak speed, and MAS values. The graph shows the relationship between FSA and the angle at peak flexion speed with participants associated with their MAS scores (see color code) using the IMU-based system. The dotted line represents the margin of the ellipse of variation computed over the control data using 2xSD. Data from both legs and days of test have been included. The abscissa shows the FSA values in degrees, and the ordinate shows the angle at peak flexion speed in degrees. The gray shaded area indicates the range of FSA values obtained in controls. The light red region includes FSA values associated only to individuals with any degree of spasticity (MAS > 0). The yellow region represents a region in which there is not complete agreement between the presence of spasticity detected by the clinical exam and FSA values. Note that this corresponds to individuals with MAS < 2

Figure 6 depicts the relationship between FSA and the angle at peak speed values obtained with the IMU-based system in controls (dark gray circles) and SCI (colored circles). FSA and angle at peak speed values were moderately correlated in controls [$r\left(78\right)=0.56, p<0.001$] and strongly correlated in individuals with SCI ($r\left(82\right)=0.87, p<0.001$). A one-way ANOVA showed a clear distinction between FSA values in individuals with MAS ≥ 2 (FSA = 54.6 ± 16.6°, 95% CI 48.6–60.6°) and individuals with no or mild spasticity (FSA = 103.0 ± 15.2°, 95% CI 96.2–106.6°, p < 0.001). Both groups were significantly different from controls (FSA = 118.1 ± 10.0°, 95% CI 114.5–121.7°, p < 0.001). A one-way ANOVA also showed that individuals with MAS ≥ 2 reached a smaller angle at peak speed (30.7 ± 9.8°, 95% CI 27.1–34.3°) compared to less spastic individuals (55.0 ± 11.0°, 95% CI 51.0–59.1°; p < 0.001) and controls (60.1 ± 8.5°, 95% CI 57.0–63.2°; p < 0.001) on both days of test. In less spastic individuals, the angle at peak speed was no different than in controls (p = 0.26). Thus, individuals could be separated in three distinct groups according to the FSA values (Fig. 6). The gray area shows the range FSA of values obtained in control participants. The light red area show the interval in which FSA values have a high agreement with the clinical exam. The upper bound of this region is represented by the smallest FSA obtained in SCI individuals identified as non-spastic by the clinical assessment (with MAS > 0). The yellow region represents an area with poor agreement between MAS scores and FSA values.

## Discussion

We compared measurements of knee extensor spasticity during the pendulum test using a 3-dimensional optical tracking system and a newly developed IMU-based system. We found no differences between FSA values obtained with the optical tracking system and the IMU-based system in control subjects and in individuals with SCI. FSA values from the IMU-based system showed excellent agreement with the optical tracking system in individuals with SCI (ICC > 0.98) and good agreement in controls (ICC > 0.82), excellent test–retest reliability across days in SCI (ICC = 0.93) and good in controls (ICC = 0.87). Notably, FSA values measured by both systems showed a moderate association with MAS scores (ρ ~ −0.8). FSA values measured by both systems were lower in individuals with SCI with spasticity compared to individuals with SCI without spasticity and control subjects. We propose that our new portable IMU-based system provides a robust and flexible alternative to a 3-dimensional optical tracking system to measure knee extensor spasticity in humans with SCI.

### Pendulum test and spasticity

The pendulum test is a widely used biomechanical test for evaluating knee extensor spasticity using kinematic analysis in humans with SCI [1, 17, 24, 25, 32, 48]. The test quantifies the effect of a gravity-induced stretch of the knee extensor muscle on the leg kinematics. A reduction of the amplitude of the first swing motion of the leg (i.e., FSA) has been associated with increased stretch reflex activity [18–21]. The pendulum test has been validated in controls [22] and has a high test–retest reliability and sensitivity to detect variations in spasticity in humans with SCI [20, 23–25], correlating with clinical scores [21, 26, 27]. In agreement with previous studies [22], in control subjects, we found mean FSA values around 114° with the optical tracking system (range = 92.2° to 132°) and around 118° with IMU-based system (range = 96.8° to 139.5°). Similarly, in participants with SCI as in other studies [32], we found FSA values around 85.3° with the optical tracking system (range = 15.9° to 128.4°) and around 84° with the IMU-based system (range = 19.9° to 134.3°). Previous work showed that the threshold velocity for activation of the stretch reflex in participants with SCI varied between 5 and 193°/s [49]. Our data shows that the knee reaches a peak speed that is 3 times greater during the pendulum test in individuals without spasticity (control: 354.5 ± 27.5°/s, SCI—mild spasticity 355.8 ± 35.2°/s), suggesting that the velocity of the movement is appropriate to elicit a stretch reflex. Moreover, in severely spastic individuals the maximum velocity of stretch occurred earlier in the flexion movement, as represented by a reduction in the angle at peak speed compared with control subjects or individuals with SCI and no spasticity. This may have resulted in smaller FSA values individuals with more severe spasticity compared to controls and less spastic individuals. Thus, the angle at peak speed could represent a useful supporting measurement to make inferences about changes in stretch reflex activation after SCI.

### Reliability of the IMU-based system

FSA values from the IMU-based system showed moderate to excellent agreement with the optical tracking system and test–retest reliability in controls and in individuals with SCI, suggesting that our new developed IMU-based system provides a robust and flexible alternative to a camera-based optical tracking system to quantify knee extensor spasticity following SCI. To ensure evaluation of reliability and specificity of the test we standardized the test procedures. For example, previous studies have shown that posture influences stretch reflex excitability and that in particular spasticity increases in the supine compared to the sitting position [19, 21, 49, 50]. Thus, we tested the FSA using the optical tracking system and IMU-based systems having subjects in the same semi-reclined position. In addition, the ability to compare the pendulum test evaluations across systems also depends on the recording equipment. Note that we found no significant discrepancies between FSA values detected by the optical tracking system and the IMU-based system in individuals with SCI. However, in control subjects, we found that on average FSA values detected by the IMU-based system were around 4.3° larger than those found by the optical tracking system. A possibility is that the small difference in FSA values detected by both systems in control subjects is related to inaccuracies in orientation estimates from the IMU sensors. Errors in estimating flexion/extension of the knee with IMUs have been shown to increase proportionally to the movement speed [41] and range [38, 40]. Note that movement speed and range were larger in controls compared to SCI participants, which can contribute to explain our results. Differences between IMU sensors and optical tracking systems in estimating knee angles during knee flexion ranged between -3 to 9.5 degrees and errors increase with movements of greater amplitude [39]. Indeed, evidence showed that IMUs can underestimate small knee flexion/extension angles and overestimate larger knee angles compared to a camera-based motion capture system reporting errors of ~ 8.0 degrees [40], which is consistent with the difference found in our study between controls and SCI participants. The fact that errors in estimation of knee joint angle are reduced with movements of smaller range, may also explain the lack of difference in the estimation of FSA values between the optical tracking and IMU-based system in individuals with SCI in our study, which achieved considerably smaller FSA during the pendulum test. Another possibility is that the larger errors we found in controls are linked to inaccuracies in estimating the position of the anatomical landmarks by the optical tracking system [34]. Evidence showed that tissue artefacts can impact markers on the thigh segment in a distal–proximal gradient adding fluctuations on knee-flexion/extension of ~ 10% of the range of motion [45].

### Clinical implications

Clinical exams used to assess knee extensor spasticity in individuals with SCI have limited repeatability and sensitivity [7–9]. The pendulum test is a biomechanical evaluation that is easy and quick to implement and requires minimal training for the operators. The ease of implementation of the test and the possibility to instrument it with a variety of commercially available sensors [51] contributed to its popularity for measuring knee extensor spasticity alongside clinical scales. To facilitate adoption in the clinics, portable sensors like IMUs, electrogoniometers, accelerometers or gyroscopes are preferred over optical tracking systems or systems based on video recordings. The higher accuracy of kinematic measurements with video-based systems comes at the cost of more expensive equipment and processing time. Moreover, accuracy may decrease due to errors in markers positioning and soft-tissue artifacts [34, 45]. However, portable sensors like electrogoniometers are sensitive to improper alignment with the joint axes [35], gyroscopes can lead to overestimation of joint angles at higher angular speeds and accuracy degrades with time, and estimations from accelerometers are affected by measurement noise and integration drift [36]. In contrast, portable sensors like IMUs have been shown to have sufficient accuracy to measure lower limb kinematics [38]. Indeed, IMUs represent a reliable and inexpensive alternative to video recordings and systems based on optical markers for estimating lower limb kinematics [37–41]. In agreement, our results support the view that our IMU-based system provides sensitive evaluation of spasticity in humans with SCI [52]. This is supported by the strong similarities in FAS values found between the IMU-based system and the optical tracking system and correlation between FSA values found between the IMU-based system and MAS scores.

A previous study found that the pendulum test was able to distinguish between spastic and non-spastic individuals [31]. Similarly, we found a clear distinction between presence (MAS ≥ 2) or absence of spasticity using the IMU-based system but in individuals classified as mildly spastic according to the clinical evaluation (MAS = 1,1 +) these estimations should be considered with caution. Based on our data, we propose that FSA ≤ 80° can be considered a conservative threshold for spasticity. However, individuals with 80° < FSA < 96.7°, which fall outside the margin of the distribution of normative data should be considered with caution. We cannot exclude the presence of larger reflex response in these individuals, as it may be present but too weak to generate enough reflex torque to significantly impact the FSA values. Importantly, the excellent test–retest reliability of the FSA values found in our study and the small within-subject variability (~ 5°), suggests that this test could be successfully used for detecting differences in spasticity following medical or therapeutic interventions longitudinally.

### Methodological considerations

It has been suggested that fast repetitions of the pendulum test may lessen spasticity measured during subsequent leg drops [18]. In our study, we had a 30 s resting period in between the 10 trails used during the pendulum test to minimize the influence of repeated muscle stretches on our outcomes. Note that we found no differences in FSA values across the 10 trials on both days, supporting the view that it is less likely that the multiple repetitions affected our results [26, 27]. This also supports the view that is it less likely the order of different testing procedures using stretching contributed to our findings. To further limit the effect of a reduction in stretch-reflex excitability due to repeated muscle stretches [26, 43], we ensured that the participants relaxed in the semi-supine position for 5 min before initiating the test, and that the MAS and the pendulum test were initiated at least 5 min apart.

IMU orientation estimates are obtained through sensor fusion algorithms that have designed to overcome some of the weaknesses of gyroscope and accelerometers sensors. However, angular estimates through IMU sensors may still suffer from non-stationarities such as drift. Even though in our study the overall effect of drift was small in relationship to the inter-trial variability of FSA measurements, we recommend limiting the test to 6 repetitions when using IMU sensors as a cautionary measure. A smaller number of repetitions appears to be sufficient to characterize spasticity with the test, given the high consistency across successive measurements in our study.

## Data Availability

The datasets used and/or analyzed during the current study are available from the corresponding author on reasonable request.
